# Furan Donor for NIR-II Molecular Fluorophores with Enhanced Bioimaging Performance

**DOI:** 10.34133/research.0039

**Published:** 2023-01-13

**Authors:** Chunchen Liu, Mengfei Li, Huilong Ma, Zhubin Hu, Xinyuan Wang, Rui Ma, Yingying Jiang, Haitao Sun, Shoujun Zhu, Yongye Liang

**Affiliations:** ^1^ Department of Materials Science and Engineering, Shenzhen Key Laboratory of Printed Organic Electronics, Southern University of Science and Technology, Shenzhen 518055, China.; ^2^ State Key Laboratory of Supramolecular Structure and Materials, College of Chemistry, Jilin University, Changchun 130012, China.; ^3^ State Key Laboratory of Precision Spectroscopy, School of Physics and Electronic Science, East China Normal University, Shanghai 200241, China.; ^4^ Department of Chemistry and Bio-X, Stanford University, Stanford, CA 94305, USA.; ^5^ Collaborative Innovation Center of Extreme Optics, Shanxi University, Taiyuan, Shanxi 030006, China.

## Abstract

The second near-infrared (NIR-II, 1,000 to 1,700 nm) molecular fluorophores containing donor–acceptor–donor conjugated backbone have attracted substantial attention due to their outstanding advantages, such as stable emission and facilely tuned photophysical properties. However, it is still challenging for them to simultaneously achieve high brightness and red-shifted absorption and emission. Herein, furan is adopted as the D unit to construct NIR-II fluorophores, demonstrating red shift of absorption, enhanced absorption coefficient, and fluorescent quantum yield when compared with the generally used thiophene counterparts. The high brightness and desirable pharmacokinetics of the optimized fluorophore, IR-FFCHP, endows improved performance for angiography and tumor-targeting imaging. Furthermore, dual-NIR-II imaging of tumor and sentinel lymph nodes (LNs) has been achieved with IR-FFCHP and PbS/CdS quantum dots, enabling the in vivo imaging navigated LN surgery in tumor-bearing mice. This work demonstrates the potential of furan for constructing bright NIR-II fluorophores for biological imaging.

## Introduction

Fluorescent imaging in the second near-infrared (NIR-II, 1,000 to 1,700 nm) region processes merits of deeper penetration and higher signal-to-background ratio (SBR) than that in the visible and first near-infrared (NIR-I, 700 to 900 nm) window [1,2]. Recently, NIR-II imaging-guided liver tumor resection surgery in human has been demonstrated [3]. Fluorophores are vital for NIR-II fluorescent imaging, but their performance is generally inferior to their NIR-I counterparts [4–13]. Organic NIR-II fluorophores, including polymethine derivatives, donor–acceptor–donor (D-A-D) conjugated molecules and A-D-A conjugated molecules, have attracted tremendous attention for angiography and tumor targeting imaging due to their good biocompatibility and easily tuned photophysical properties [6,14–16]. It has been recently reported that organic fluorophores can be successfully utilized for multiplexed NIR-II imaging of sophisticated metastasis progress from tumor to sentinel lymph node (LN) [17]. To achieve improved bioimaging performance, fluorophores are expected to possess high brightness and longer absorption and emission wavelength [1–3,15,18].

Thus far, D-A-D fluorophores have been extensively studied because of their outstanding photostability and facilely tuned absorption and emission properties. Notably, a shielding unit (S, such as alkyl chain-substituted fluorene) could be introduced to weaken intermolecular interactions among conjugated backbones, resulting in improved fluorescence quantum yields (QYs) for S-D-A-D-S fluorophores [19,20]. It has also been demonstrated that rational engineering of the D unit can induce substantial tuning on the optical properties and pharmacokinetics of these fluorophores [14,21–25]. The steric size and hydrophobicity of D unit can effectively alter the backbone geometry and intermolecular interaction, which are closely correlated with the absorption or emission wavelength, QY, and absorption coefficient (*ε*) of fluorophores. Up to date, the D unit manipulation is generally focused on the thiophene derivatives, and considerable achievements have been realized [20,21,24]. For instance, replacing the thiophene (T) donor with 3,4-ethylenedioxy thiophene can result in conjugated backbone distortion, which can protect the A unit in the excited state and eventually afford QY enhancement. Furthermore, using alkyl thiophene as the D unit can enhance the hydrophobicity of molecules and decrease the interaction between acceptor unit and surrounding water, resulting in improved QYs in aqueous solutions [24]. However, alkyl thiophene introduction could adversely afford blue shift of absorption and emission wavelength [26–29]. Therefore, it is urgent to find new donor units to construct highly fluorescent NIR-II fluorophores without blue-shifting the absorption wavelength.

Herein, structure optimization is adopted on the donor moiety of S-D-A-D-S fluorophores, where the furan donor is first adopted for NIR-II fluorophores because of its stronger electron donating ability than thiophene unit. The fluorophores with furan donor exhibit about 80-nm red shift of absorption and a substantial increase of *ε* compared to IR-FA with a thiophene donor [24]. Further, the side chains on furan donor moiety are delicately designed from *n*-octyl to 2-cyclohexylmethyl chain, forming fluorophores IR-FFC8P and IR-FFCHP, respectively. IR-FFCHP with a cyclohexyl-methyl furan donor exhibits a QY of 0.73% (QY = 0.05% of IR-26 in ethylene dichloride as the reference), which is the highest reported value for S-D-A-D-S fluorophores. Theoretical calculation results reveal that the cyclohexyl–methyl side chain can enhance protection of backbone from interaction with water without increasing molecular backbone distortion. The high brightness makes IR-FFCHP competent for in vivo whole-body angiography in mice with high resolution. Combining with PbS/CdS core/shell quantum dots (QDs) [30], dual-colored NIR-II imaging has been achieved through locating the tumor with IR-FFCHP in a NIR-IIa (1,000 to 1,300 nm) channel while mapping the sentinel LNs with QDs in a NIR-IIb (1,500 to 1,700 nm) channel, eventually enabling precise resection of sentinel LNs under high imaging contrast and depth.

## Results and Discussion

### Fluorophore design

**Scheme. 1.**
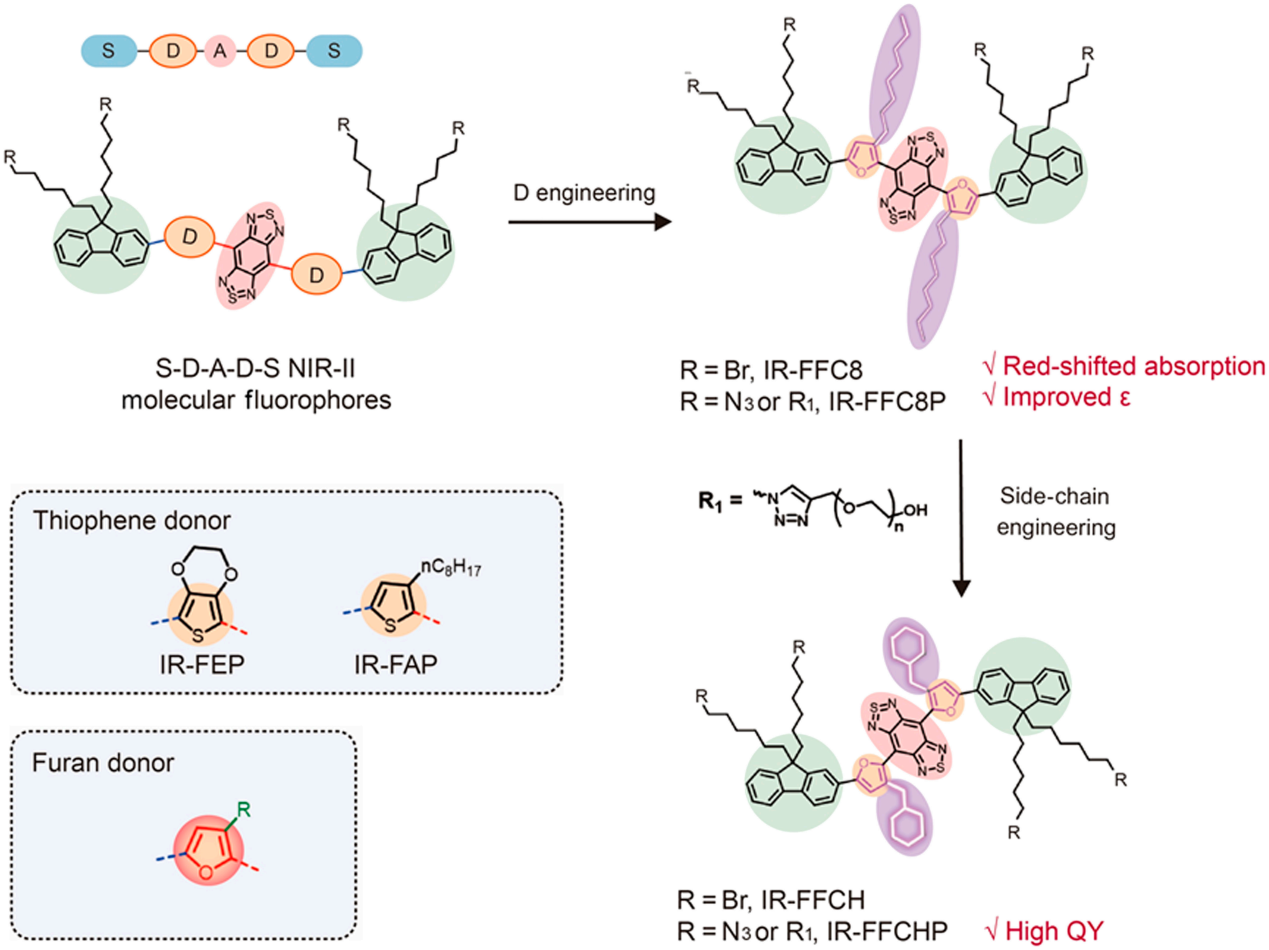
Structural illustration of S-D-A-D-S fluorophores and structural regulation of molecular fluorophores in this work.

As shown in Scheme 1, a strong electron-deficient unit, benzo[1,2-c:4,5-c’]bis[1,2,5]thiadiazole (BBTD), is adopted as the A unit to induce a large intramolecular charger transfer effect, affording absorption and emission at a long-wavelength region [31]. Fluorene substituted with di-octyl chains is used as the shielding unit, whereas the generally used thiophene donor is replaced with the furan unit, yielding molecules IR-FFC8 and IR-FFCH. In order to enhance solubility in aqueous solutions, polyethylene glycol (PEG) chains are linked at fluorene side chain terminal through a click reaction, affording the water-soluble fluorophores IR-FFC8P and IR-FFCHP. The smaller size of oxygen atom than the sulfur atom and the stronger electron donating ability of furan are speculated to endow the fluorophores with red-shifted absorption. It is noteworthy that the 3-dimensional extending cyclohexyl-methyl chain is adopted to replace the *n*-octyl chain in IR-FFCH in order to substantially decrease the interaction between the conjugated backbone and the surrounding water molecules. To the best of our knowledge, it is the first attempt to adopt cyclo-alkyl side chains for NIR-II molecular fluorophores. The synthesis details of fluorophores are shown in the Supplementary Materials.

### Photophysical properties

**Fig. 1.**
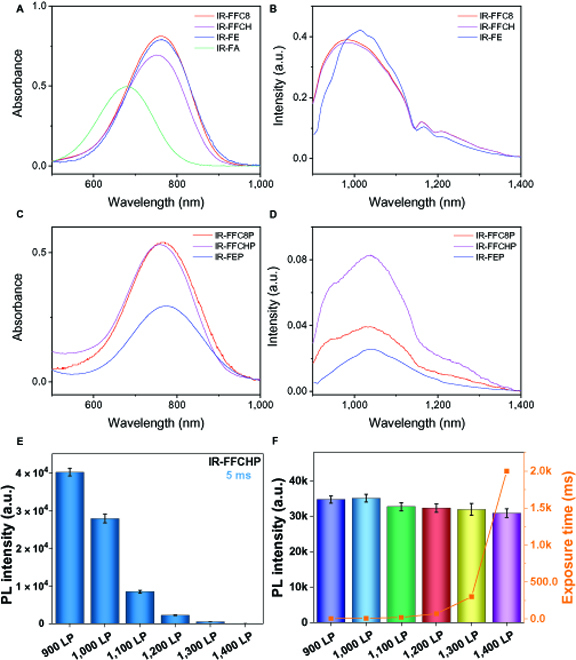
Optical properties of the synthesized fluorophores. Absorption (A) and emission (B) properties of the un-PEGylated fluorophores in toluene. Absorption (C) and emission (D) properties of the PEGylated fluorophores in deionized water. Measured concentration: 4.0 × 10^−5^ M, excitation: 808-nm laser. Photoluminescence (PL) intensity at varied long-pass (LP) wavelength (900 to 1,400 nm) for IR-FFCHP with a low concentration in phosphate buffer saline (0.5 μM) under irradiation of a 65 mW/cm^2^ laser for 5 ms (E) and increasing exposure time (F). a.u., arbitrary units.

The optical properties of un-PEGylated fluorophores IR-FFC8 and IR-FFCH were first investigated via absorption and emission spectra in toluene (Fig. 1A and B and Table). Two fluorophores exhibit similar absorption peaks at about 760 nm and these are 80 nm red-shifted when compared with the thiophene counterpart IR-FA [24]. The maximum absorption coefficient (*ε*) of IR-FFC8 is 20.0 × 10^3^ M^−1^·cm^−1^, slightly higher than 17.0 × 10^3^ M^−1^·cm^−1^ of IR-FFCH, both of which are higher than 12.0 × 10^3^ M^−1^·cm^−1^ of IR-FA with the thiophene donor. Similar emission spectra with a peak at 980 to 1,010 nm and a comparable QY of ~3.0% are determined for these fluorophores in toluene.

**Table.** Optical parameters of the fluorophores. QE (brightness value) = QY × *ε*. The emission of IR-FA was not measured because of its negligible absorption at 808 nm.

Fluorophore	Solvent	*ε*_max_ (10^3^ M^−1^·cm^−1^)	*λ*_abs_ (nm)	*λ*_em_ (nm)	Stokes shift (nm)	*Ф*_f_ (%)	QE
IR-FE^[^^20^^]^	Toluene	19	767	1,013	246	3.1	487
IR-FA^[^^24^^]^		12	680	NA	NA	N/A	NA
IR-FFC8		20.0	765	981	216	2.8	560
IR-FFCH		17.0	752	982	230	2.7	459
IR-FEP^[^^20^^]^	Water	5.7	780	1,047	267	0.2	11
IR-FFC8P		12.7	766	1,044	278	0.44	56
IR-FFCHP		12.5	760	1,038	278	0.73	91

N/A, not applicable

As shown in Fig. 1C and D and Table, in the aqueous environment, 2 PEGylated fluorophores, IR-FFC8P and IR-FFCHP, show similar absorption properties with a peak at ~760 nm and *ε* of ~12.5 × 10^3^ M^−1^·cm^−1^, over 2-fold higher than that of IR-FEP (5.7 × 10^3^ M^−1^·cm^−1^) [24]. Impressively, IR-FFCHP (QY = 0.73%) shows much stronger fluorescent emission than IR-FEP (QY = 0.20%). It is among the highest value of the state-of-the-art QYs for S-D-A-D-S fluorophores soluble in aqueous solutions. The record high QY and *ε* together afford IR-FFCHP with fluorescence brightness value of 91 in water, outperforming IR-FEP [24]. Figure 1E and F illustrates the photoluminescence (PL) intensity at long-pass (LP) wavelength varying from 900 to 1,400 nm for IR-FFCHP with a low concentration in phosphate buffer saline (0.5 μM) under irradiation of an 808-nm laser. Under a high-speed exposure (5 ms), IR-FFCHP aqueous solution exhibits an outstanding PL intensity at 900-nm LP, and it decreases with increasing the LP wavelength to 1,400 nm (Fig. 1E). The fluorescence at 1,300-nm LP still could be observed presumably because of the high brightness of IR-FFCHP. With increasing the exposure time, the IR-FFCHP aqueous solution can display a comparable PL intensity at even 1,400-nm LP (Fig. 1F). Particularly, at such a low concentration (0.5 μM) and low laser power (65 mW/cm^2^), comparable PL intensity at 1,300 LP can be realized with 300 ms of exposure, indicating the state-of-the-art PL performance of IR-FFCHP for water-soluble D-A-D fluorophores reported to date. The photostability of 2 fluorophores was measured under continuous laser irradiation (808 nm) for 140 min, and both fluorophores show excellent stability when compared with indocyanine green (ICG) and IR-800 CW (Fig. S1). Both fluorophores exhibit small sizes of about 10 nm from the dynamic light scattering measurement (Fig. S2).

### Theoretical simulation

Firstly, the density functional theory and time-dependent density functional theory calculations were conducted to investigate molecular geometries, and optical and electronic properties of fluorophores are gained with the optimally tuned *ω*B97XD*/6-31G(d) method (see computational details in the Supplementary Materials) [24,32,33]. It is observed that both the 2 fluorophores possess similar electronic structures and energy levels accompanying with the whole backbones-delocalized highest occupied molecular orbitals (~ −5.80 eV) and the BBTD-localized lowest unoccupied molecular orbitals (~ −4.40 eV) (Fig. S3). The corresponding highest occupied molecular orbitals are obviously higher than that of IR-FTA (−6.20 eV), while the lowest unoccupied molecular orbitals remain almost unchanged when compared to that of IR-FTA (−4.50 eV) [21], confirming the stronger electron donating ability of furan than of thiophene unit. Additionally, these 2 fluorophores exhibit almost identical molecular backbone distortion for ground state (*S*_0_) as well as the first singlet excited state (*S*_1_) (Fig. S4). It is noteworthy that the dihedral angles between central BBTD and furan unit at *S*_o_ and *S*_1_ states are calculated to be 42^o^ and 37^o^, respectively, suggesting a more delocalized electronic structure of the *S*_1_ excited state vs. the *S*_0_ ground state. Both of them are smaller than the angles between BBTD and thiophene unit of IR-FTA (58^o^ and 40^o^) [21]; the smaller distortion effect can be attributed to the smaller atom size of oxygen than sulfur, resulting in the red-shifted absorption and emission of the designed fluorophores. However, the similar electronic structures for both IR-FFC8 and IR-FFCH prompt further consideration of their states under the aqueous-solution condition.

**Fig. 2.**
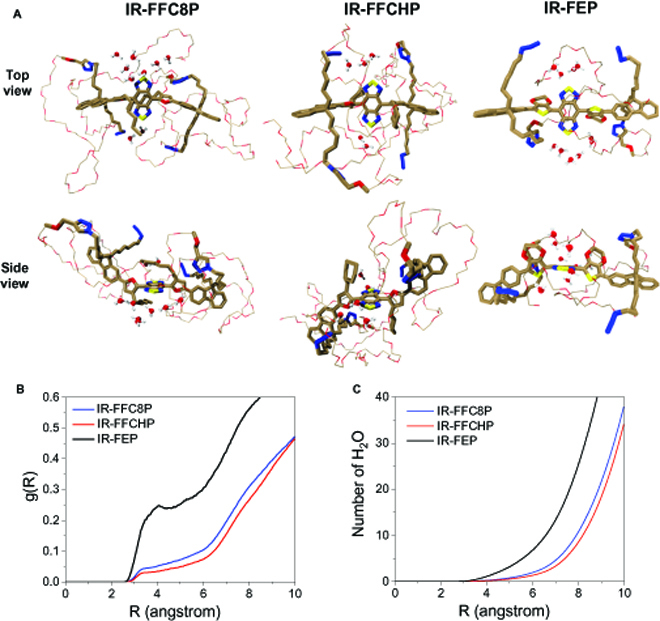
Molecular dynamic simulations of the fluorophores. (A) Schematic diagram of interaction between H_2_O and fluorophores in aqueous circumstances through molecular dynamic simulations. Brown, red, yellow, blue, and thin line parts with gray and red represent the C, O, S, N, and PEG chain, respectively. (B) RDF of O atoms from water around the BBTD of fluorophores with the radius (angstrom) around BBTD core. (C) Counted H_2_O number surrounding BBTD.

Therefore, molecular dynamic simulations were further conducted to investigate the structural evolution of the 2 fluorophores in aqueous solutions and their interactions with surrounding water molecules [16]. Note that water has been demonstrated to reduce the fluorescence QY by more than 2 orders of magnitude and acts as an efficient fluorescence quencher for NIR-II fluorophores [21,34]. As shown in Fig. 2A, because of the alkyl chain, better molecular core protection from interaction with water molecules can be achieved for IR-FFCHP and IR-FFC8P when compared with IR-FEP. The radial distribution function (RDF) of H_2_O molecules and counted H_2_O number are presented in Fig. 2B and C, respectively. Both IR-FFCHP and IR-FFC8P display obviously smaller RDF values, suggesting less H_2_O molecules encompassing the BBTD center than IR-FEP. It may mainly account for the higher QYs and ε of IR-FFC8P and IR-FFCHP than IR-FEP. Particularly, when compared with IR-FFC8P, benefiting from the more substantial steric hindrance of cyclohexyl group, smaller RDF and decreased water-molecule numbers can be observed for IR-FFCHP. Such a larger 3-dimensional size of cyclohexyl group can afford better protection than the *n*-octyl chain on BBTD acceptor unit as well as the whole molecular backbone from the unfavorable quenching interactions with surrounding H_2_O molecules, thus endowing IR-FFCHP with a higher QY [21].

### In vivo NIR-II angiography

Real-time and high-resolution angiography through a noninvasive route is helpful for studying microscopic biological processes. Current NIR angiography modality based on ICG contrast agent suffers from low imaging quality/depth and limited imaging window. The fluorophore IR-FFCHP with higher brightness was first utilized for in vivo bioimaging of vascular networks in mice. Prior to the administration of fluorophore on mice, the biocompatibility of IR-FFCHP was evaluated. The hemolysis test was conducted, and the result reveals that IR-FFCHP exhibits good blood compatibility (Fig. S5). Additionally, the cellular toxicity of IR-FFCHP was measured through standard methylthiazolyldiphenyl-tetrazolium assays, and results indicate that more than 90% of cells survived after incubation (24 h) with IR-FFCHP at a concentration up to 20 μg/ml (Fig. S6). Collectively, IR-FFCHP shows low biotoxicity.

**Fig. 3.**
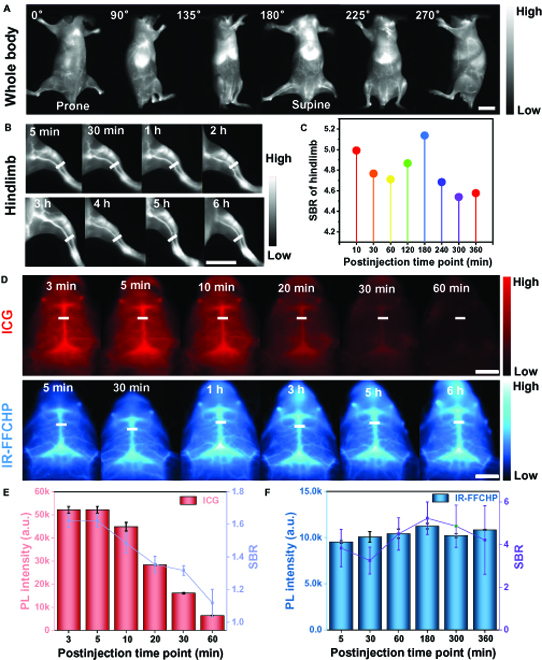
Imaging performance of the fluorophores. Vessels imaging for the whole-body (A) and magnified hindlimb (B). (C) The signal-to-background ratio (SBR) of the specified position of imaged hindlimb at different post-injection time points. (D) Brain vessel imaging in mice treated with indocyanine green (ICG) and IR-FFCHP. The PL intensity and SBR changes of marked position on brain vascular for ICG (E) and IR-FFCHP (F) with different post-injection time points. Intravenous injection dose: 100 μl, 1 mg/ml; LP filter: 1,200 nm; exposure time: 200 ms; excitation: 808-nm laser (80 mW/cm^2^). The scale bars represent 1 cm.

In Fig. 3A, the whole-body vascular imaging was initially performed, and it is found that blood vessels can be clearly figured out at various positions of the mouse. After magnifying the imaging region, sophisticated vessels network in different body parts, such as back, belly, hindlimb, and foot, can be clearly discriminated (Fig. S7). The hindlimb vascular system was further scrutinized under an LP filter of 1,200 nm at different time points (Fig. 3B). High-quality imaging of hindlimb vessels is able to be achieved at 5 min post-injection of IR-FFCHP, and considerable imaging quality can be maintained even at 6 h after injection, suggesting its long blood circulation. Figure 3C plots the SBR of the specified position of imaged hindlimb at different post-injection time points; it can be observed that highest SBR of 5.2 can be achieved at 3 h after injection. As shown in Fig. 3D, brain vessels in mice treated with ICG cannot be observed at 60 min after injection, while they can be observable for the mice treated with IR-FFCHP even at 6 h after injection, further demonstrating the long-term imaging ability of IR-FFCHP. The PL intensity and SBR changes of marked position on brain vessels for ICG and IR-FFCHP with different post-injection time points were presented in Fig. 3E and F. It can be clearly observed that the PL intensity and SBR are dramatically reduced with prolonging the post-injection time for ICG-treated mice, while SBR remains almost consistent (SBR ≈ 4) for IR-FFCHP-treated mice at even 6 h after injection. The imaging quality of cerebral and hindlimb vessels was also evaluated under different LP filters (1,000 to 1,300 nm) and exposure time (10 to 700 ms) (Figs. S8 and S9). Benefiting from the high brightness and long emission wavelength of IR-FFCHP, the tissue autofluorescence, absorption, and scattering are substantially decreased at longer wavelength region; therefore, it is found that imaging under longer LP filters can get more enhanced resolution improvement.

### Pharmacokinetics and tumor-targeting imaging

**Fig. 4.**
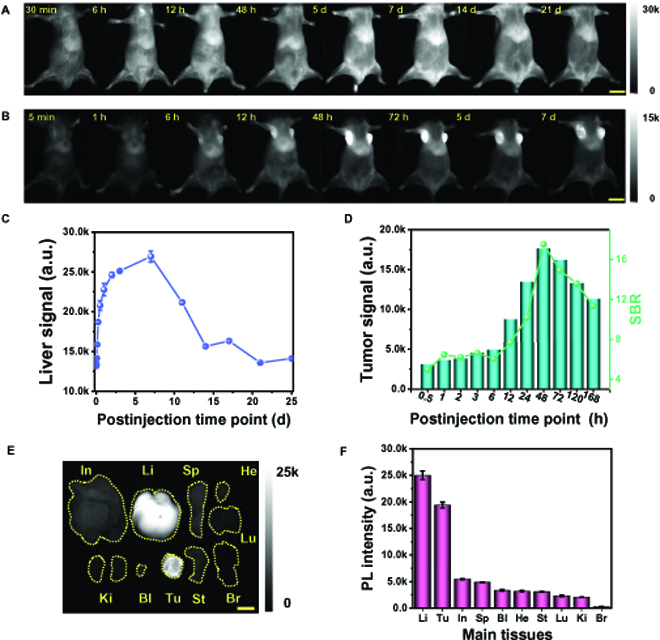
Pharmacokinetics and targeted imaging of 4T1 tumor model with IR-FFCHP. (A) In vivo circulation of IR-FFCHP. (B) Imaging of a mouse with 4T1 tumor after intravenous injection of IR-FFCHP; the high EPR effect on 4T1 tumor is observed because of the long circulation of fluorophores. The corresponding fluorescence signal intensity of liver (C) and tumor (D) as a function of time. (E) Ex vivo imaging of main organs of IR-FFCHP-treated mice after 7 d post-injection. (F) The fluorescence intensity values of main organs of IR-FFCHP treated mice. Imaging condition: 1,200-nm LP, 100-ms exposure time, 80 mW/cm^2^ laser power. The scale bars represent 1 cm. In, intestine; Li, liver; Sp, spleen; He, heart; Lu, lung; Ki, kidney; Bl, bladder; Tu, tumor; St, stomach; Br, brain.

After intravenous injection of IR-FFCHP into mice, the accumulation of fluorophores is mainly observed in the liver (Fig. 4A and B). Liver fluorescence signal reaches the peak at 7 d post-injection and gradually decreases to below the detectable level at 25 d post-injection (Fig. 4C), demonstrating the hepatobiliary excretion pathway of IR-FFCHP in mice. After injecting IR-FFCHP in 4T1 tumor-bearing mice, the long blood circulation of IR-FFCHP enables effective passive tumor targeting ability (Fig. 4B). The tumor signal gradually increases from 5 min post-injection of IR-FFCHP and reaches the peak at 48 h, which subsequently decreases until 7 d post-injection (Fig. 4D). It is plausible that the long circulation of IR-FFCHP in mice can improve the enhanced permeability and retention (EPR) effect and lead to high-quality tumor-targeting imaging [35]. The biodistribution of IR-FFCHP in different organs was further investigated through the imaging of surgically separated organs, and the result reveals that the fluorophore is mainly accumulated in liver and tumor (Fig. 4E and F), once again demonstrating the hepatobiliary metabolization pathway and EPR-assisted tumor accumulation of IR-FFCHP.

### Dual-colored NIR-II imaging-guided surgery

**Fig. 5.**
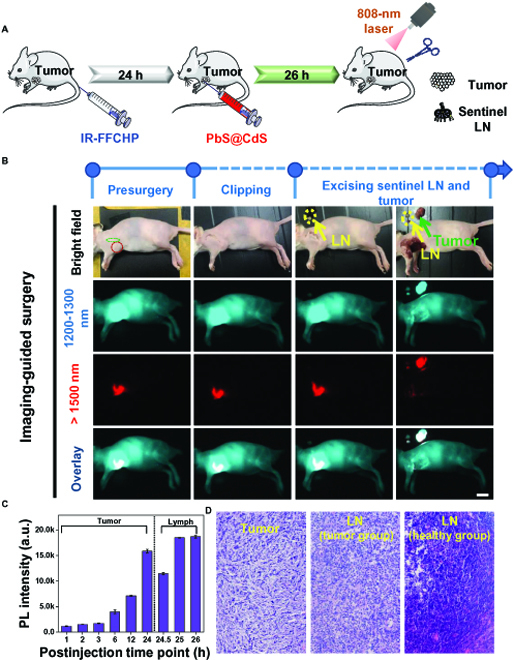
NIR-II imaging guided surgery with IR-FFCHP. (A) The schematic illustration of dual-colored in vivo NIR-II imaging guided 4T1 tumor-sentinel LN surgery. (B) Tumor imaging with IR-FFCHP in 1,200- to 1,300-nm window and sentinel LN imaging with QDs in >1,500-nm window. (C) PL intensity of tumor and LN at different time points after sequential injection of IR-FFCHP and QDs. (D) H&E stain of the resected tumor and LNs with/without tumor metastasis. The scale bar represents 1 cm.

Resection of tumor tissues is the main strategy for cancer treatment. Considering that the sentinel LNs are the principal pathway in tumor metastasis, which is closely related with tumor prognosis, tumor staging identification, and treatment decision [35], it is of vital importance to concurrently visualize the metastasis of tumor in sentinel LNs during the imaging navigated tumor excision in clinical applications [35]. Here, we used the IR-FFCHP for tumor NIR-IIa (1,000 to 1,500 nm) imaging because of its high fluorescence brightness and intense retention in tumor. In order to achieve dual-colored imaging, PbS/CdS QDs were utilized for NIR-IIb (1,500 to 1,700 nm) imaging of tumor-associated sentinel LNs [30]. We initially attempted to investigate the NIR-IIb imaging of the QDs for mouse hindlimb LNs (Fig. S10). Two mice footpads were intradermally injected with IR-FFCHP and QDs, respectively. It can be observed that the signal of IR-FFCHP cannot be detected with an LP filter of 1,500 nm, indicating the nonoverlapping dual-colored imaging between sentinel LNs using QDs and tumor with IR-FFCHP. Therefore, dual-colored bioimaging can be successfully conducted. At 24 h post-administration of IR-FFCHP, the QDs were intratumorally injected, and the tumor and LN signals can both reach the optimal intensity at 26 h post-injection (Fig. 5A and C and Fig. S11). The tumor can be figured out within an NIR-IIa window, while the cancer-associated LNs can be discriminated in an NIR-IIb window, respectively. With the help of dual-colored imaging, the resection surgery of tumor and sentinel LNs was successfully accomplished (Fig. 5B). The hematoxylin and eosin (H&E) stain of the resected tumor and LNs with/without tumor metastasis is illustrated in Fig. 5D, and the result further demonstrates the accomplishment of precise resection surgery under dual-colored NIR-II imaging. Additionally, organs in normal and 4T1 tumor-bearing mice were also treated with the H&E stain method, and the results verify the in vivo biocompatibility of IR-FFCHP (Fig. S12).

## Conclusion

In summary, furan is adopted for the first time as the D unit to construct 2 S-D-A-D-S fluorophores, IR-FFC8P and IR-FFCHP. Compared to thiophene counterparts, stronger electron donating ability and smaller size of oxygen atom together endow the furan fluorophores with a stronger intramolecular charger transfer effect and less molecular backbone distortion, resulting in red-shifted absorption and enhanced absorption coefficient. The Cyclohexyl–methyl side chain on a furan donor can afford better protection on a BBTD unit from unfavorable quenching interaction with surrounding water molecules without increasing conjugated backbone distortion, affording IR-FFCHP with the record-high QY of 0.73% for reported S-D-A-D-S structural fluorophores. In addition to high brightness, IR-FFCHP also exhibits long blood circulation in mice, enabling better brain vascular imaging than clinical ICG and intense tumor retention. Combined with NIR-IIb QDs, dual-colored NIR-II imaging using IR-FFCHP to mark tumors and QDs to distinguish sentinel LNs is demonstrated, which enables the in vivo imaging-navigated surgery of LNs in 4T1 tumor-bearing mice. This work provides new insights for developing high-performance NIR-II molecular fluorophores through delicate molecular engineering strategies, which is expected to accelerate the application of NIR-II fluorophores in sophisticated in vivo imaging. This furan donor modification strategy can also be utilized for constructing donor–acceptor conjugated polymers with strong absorption in NIR-II windows and high photothermal conversion efficiency for deep-brain neuromodulation and other related applications [36].

## Data Availability

The data used to support the findings of this study are available from the corresponding authors upon request.
