# Supplementary material for: Furan Donor for NIR-II Molecular Fluorophores with Enhanced Bioimaging Performance
Source: Research (Wash D C). 2023 Jan 13;6:0039. doi: 10.34133/research.0039 (PMC10076007; doi:10.34133/research.0039)
Supplement: Supplementary Materials — Methods. Figs. S1 to S12. Tables S1 and S2. [file research.0039.f1.docx]

***Supplementary Materials***

**Furan Donor for NIR-II Molecular Fluorophores with Enhanced Bioimaging Performance**

Chunchen Liu,^1^ Mengfei Li,^2^ Huilong Ma,^1^ Zhubin Hu^3^, Xinyuan Wang,^1^ Rui Ma,^1^ Yingying Jiang,^4^ Haitao Sun,^3,5*^ Shoujun Zhu^2^ and Yongye Liang^1^*

^1^Department of Materials Science and Engineering, Shenzhen Key Laboratory of Printed Organic Electronics, Southern University of Science and Technology, Shenzhen 518055, China.

^2^State Key Laboratory of Supramolecular Structure and Materials, College of Chemistry, Jilin University, Changchun 130012, China.

^3^State Key Laboratory of Precision Spectroscopy, School of Physics and Electronic Science, East China Normal University, Shanghai 200241, China.

^4^Department of Chemistry, Stanford University, Stanford, CA 94305; Bio-X, Stanford University, Stanford, CA 94305

^5^Collaborative Innovation Center of Extreme Optics, Shanxi University, Taiyuan, Shanxi, 030006, China

*Correspondence:

Yongye Liang, [liangyy@sustech.edu.cn](mailto:liangyy@sustech.edu.cn)

Haitao Sun, [htsun@phy.ecnu.edu.cn](mailto:htsun@phy.ecnu.edu.cn)

Shoujun Zhu, [sjzhu@jlu.edu.cn](mailto:sjzhu@jlu.edu.cn)

**1. Methods**

**Materials.** Unless otherwise noted, all reagents were obtained commercially and used without further purification. Tetrahydrofuran (THF), toluene, and dichloromethane (DCM) used for reactions were purified by a solvent purification system (Innovative Technology, Inc.) before using. All air and moisture sensitive reactions were carried out in flame-dried glass-wares under a nitrogen atmosphere.

**General measurements.** ^1^H and ^13^C NMR spectra were performed on 500 MHz and 400 MHz NMR spectrometers (Bruker AVANCE). Mass spectra were in general recorded on QSTAR Elite (ABI). Ultraviolet-visible-near infrared (UV-VIS-NIR) absorption spectra were recorded on Shimadzu UV-3600Plus. Size exclusion chromatography (SEC) was performed on Malvern Viscotek 270 max with 10 μm PLgel 600 × 7.5 mm column. THF was used as the mobile phase at a flow rate of 1.0 mL/min at 40 ^◦^C.

***Fluorescence Quantum Yield Measurement.*** The fluorescence quantum yields of the fluorophores were measured in a similar way to a previous method. [20] The fluorescence spectra in the region of 900-1500 nm were measured by a spectrometer with a thermoelectrically cooled InGaAs detector (HORIBA Ihr320) under an 808 nm diode laser excitation (RMPC lasers, 180 mW). During emission measurements, one 850-nm short pass filter (Thorlabs) was used as the emission filter. The obtained emission spectra were further corrected by the detector sensitivity profile and the absorbance features of the filter. The fluorescence quantum yield was determined against the reference fluorophore IR-FE with a known quantum yield of 3.1% (*Φ*_st_) in toluene, which was previously determined with IR-26 of 0.050% as reference in dichloroethane. All samples were measured at 25 ^o^C with optical density (*OD*) below 0.1 at 808 nm. The intensity read out from the InGaAs camera was a spectrally integrated total emission intensity in the 900-1400 nm region. Using the measured optical density (*OD*) at 808 nm and spectrally integrated fluorescence intensity (*F*), the quantum yield of the test sample can be calculated according to the following equation:

$$Ф_{x}\left( \lambda\right)= Ф_{st}\left( \lambda\right) \times\frac{F_{x}}{F_{st}} \times\frac{A_{st} \left( \lambda\right)}{A_{x} \left( \lambda\right)}\times\frac{\eta_{x}^{2}}{\eta_{st}^{2}}= Ф_{st}\left( \lambda\right) \times\frac{F_{x}}{F_{st}} \times\frac{1 - {10}^{-{{OD}_{st} \left( \lambda\right)}}}{1 - {10}^{-{{OD}_{x} \left( \lambda\right)}}}\times\frac{\eta_{x}^{2}}{\eta_{st}^{2}}$$

*Φ*_st_ and *F*_st_ are data of the IR-FE standard, *Φ*_x_ and *F*_x_ are data of the studied sample. η is the refractive index of solvent.

***Animal Experiments.*** Animal experiments were approved by the Institute of Radiation Medicine, Chinese Academy of Medical Sciences administrative panel on Laboratory Animal Care. All animal experiments in this work were handled under protocols approved by the Institutional Animal Care and Use Committee of Jilin University (Protocol number: 20210642). C57BL/6 and Balb/C mice were purchased from Liaoning Changsheng Biotechnology Co., Ltd. Bedding, nesting materials, food and water were provided ad libitum. Ambient temperature was controlled at 20 to 22°C with 12-hour light/12-hour dark cycles.

***In vivo NIR-II Fluorescence Imaging.*** NIR-II fluorescence images were collected using a thermoelectrically cooled, two-dimensional InGaAs array (Princeton Instruments, 640 × 512 pixels). The excitation light was provided by a fiber-coupled 808-nm diode laser (RMPC) with an in-plane excitation power density of 80 mW/cm^2^. The light was collimated and filtered through a 4.5 mm collimator and an 850-nm and a 1000-nm short pass filter (Thorlabs). The emission light was filtered using a 1000-nm, 1200-nm, 1300-nm and 1500-nm long pass filter (Thorlabs), and focused onto the detector.

***Molecular dynamic simulations.*** The initial ground-state structure was optimized with Gaussian 16 program^[1]^ at the PCM(water)-B3LYP/6-311G(d,p) level^[2]^, and then the restrained electrostatic potential (RESP) charges^[3]^ and the General Amber Force Field (GAFF)^[4]^ were assigned for the optimized structure. Thus the structure was immersed in the center of a truncated octahedral box of TIP3P water^[5]^ molecules, and all of the peptide atoms were no less than 8 Å from the boundary of the water box. And 2000 steps of steepest descent followed by 8000 steps of conjugate gradient energy minimizations were performed to remove bad contacts before the simulation. The relaxed structure was then gently heated from 0 to 300 K in 50 ps and equilibrated for 50 ps with weak restraints on each molecule, which was equilibrated for another 500 ps at constant pressure without restraint. Production simulations were extended to 300 ns for each molecule and trajectories were saved every 2 ps. All bonds with hydrogen atoms were fixed using the SHAKE algorithm^[6]^. The particle mesh Ewald method with an 8 Å cutoff in real space was used to calculate electrostatic interaction. A Langevin thermostat with a collision frequency of 1.0 𝑝𝑠^−1^ was used to regulate temperature. Isotropic pressure coupling with a relaxation time of 2 ps was used to maintain the pressure to 1 atm. All the MD simulations were performed by Amber 18 program^[7]^.

***Quantum Chemical Calculations:*** To reduce the computational cost, alkyl substituent groups on fluorene units were replaced by methyl groups. The ground-state (S_0_) geometries of simplified structures **IR-FFC8** and **IR-FFMCH** were firstly optimized using density functional theory (DFT) at the B3LYP-D3BJ/6-31G(d) level and re-optimized at the optimally-tuned-*ω*B97XD*/6-31G(d) level^[9]^. The corresponding range separation parameter (*ω*, in Bohr^-1^) for each molecule was optimally tuned using “GAP-Tuning” procedure^[10-11]^ and listed in **Supplementary Table 1**. The excited-state (S_1_) geometries of these molecules were optimized using time dependent (TD)-*ω*B97XD*/6-31G(d) method. The HOMO and LUMO orbitals, absorption excitation energies of these molecules were obtained at the TD-*ω*B97XD*/6-31G(d) level based on their corresponding S_0_ geometries. The emission energies of these molecules were calculated at the TD-tuned-*ω*B97XD*/6-31G(d) level based on their corresponding optimized S_1_ geometries. All the DFT and TDDFT calculations were performed using the Gaussian 16 program.

**2. Fluorophores synthesis and characterization.**

**Synthesis of IR-FFC8P**

**Scheme S1.** The synthetic routes for IR-FFC8P. Reagents and conditions: a) n-BuLi, THF, -78˚C to RT, argon, 1h; TIPSCl, argon, 0 ˚C to RT, 12h, 86%; b) n-BuLi, THF, -78˚C to RT, argon, 1h; n-Bu_3_SnCl, argon, 0 ˚C to RT, 12h; c) toluene, Pd (PPh_3_)_4_, argon, reflux, overnight, 71%; d) TFA, DCM, RT, 1h, 83%; e) n-BuLi, THF, -78˚C to RT, argon, 1h; n-Bu_3_SnCl, argon, 0 ˚C to RT, 12h; f) BBTD-Br_2_, Pd (PPh_3_)_4_, toluene, reflux, overnight, 39%; g) NaN_3_,DMF, RT, 3h; h) TcCu, TBTA, w-alkynyl-PEG-hydroxyl PEG1500 (Mn = 1500), THF, RT, 30 min, 81%.

**Compound 1.** The compound 1 was prepared according to reported procedures [3].

**Compound 2.** To solution of compound 1 (1.8 g, 10 mmol) in 40 mL THF, 6.56 mL n-BuLi (1.6 M, 1.05 eq) was added at –78 ᵒC under argon. After stirred 1h at room temperature, TIPSCl (2.12 g, 11 mmol, 1.1 eq) was added at 0 ᵒC. After stirred 12 h at room temperature, it was quenched by sat. NH_4_Cl. Then the mixture was extracted by petroleum ether. The organic phase was dried by MgSO_4_ and concentrated in vacuum. The residue was purified on silica column to afford compound as colorless oil (2.9g, 86%). ^1^H-NMR (CDCl_3_ 500 MHz) 0.88 (3H, t, J=6.83 Hz), 1.08 (18H, d, J=7.45 Hz), 1.27-1.38 (15H, m), 2.45 (2H, t, J=8.03 Hz), 6.31 (1H, s), 7.57 (1H, s). ^13^C-NMR (CDCl_3_ 126 MHz) 14.31, 17.71, 22.83, 22.41, 24.89, 29.41, 29.54, 29.63, 30.21, 32.02, 107.86, 119.78, 141.09, 157.34. ESI-MS: [M+H]^+^ 337.3.

**Compound 3.** To a solution of compound 2 (1.35 g, 4.0 mmol) in 40 mL THF, 1.92 mL 2.5 M n-BuLi (4.8 mmol, 1.2 eq) was added at –78 ᵒC under argon. After stirred 1h at room temperature, Bu_3_SnCl (2.1 g, 6.0 mmol, 1.5 eq) was added at 0ᵒC. After stirred 12h at room temperature, the reaction was quenched by water and extracted by EA. The organic phase was dried by Na_2_SO_4_ and concentrated in vacuum to afford compound 3, which was used in next step without purification.

**Compound 4.** The solution of above given compound 3, 2-bromo-9,9-bis(6-bromohexyl)-9H-fluorene (2.28 g, 4.42 mmol, 1 eq), Pd(PPh_3_)_4_ (200 mg, 0.22 mmol, 0.05 eq) in 50 mL toluene was refluxed overnight under argon. The solvent was removed in vacuum and the residue was purified by silica column to afford compound 4 was white solid (2.35 g, 71%). ^1^H-NMR (CDCl_3_ 500 MHz) 0.86 (3H, t, J=6.78 Hz), 1.15-1.20 (21H, m), 1.27-1.33 (14H, m), 1.41-1.46 (8H, m), 1.65 (6H, quint, J=7.13 Hz), 1.98 (4H, t, J=7.78 Hz), 2.50 (4H, t, J=8.03 Hz), 3.26 (4H, t, J=6.83 Hz), 6.68 (1H, s), 7.27-7.34 (3H, m), 7.59 (1H, s), 7.66-7.70 (3H, m). ^13^C-NMR (CDCl_3_, 126 MHz) 14.29, 18.97, 22.85, 23.66, 26.44, 27.05, 27.89, 29.22, 29.42, 29.67, 30.05, 31.16, 31.74, 32.05, 32.80, 34.09, 40.40, 55.01, 106.72, 118.12, 119.82, 120.12, 122.85, 122.93, 127.05, 127.15, 130.39, 139.44, 140.31, 140.98, 150.67, 150.85, 151.32, 157.87. ESI-MS: [M+H]^+^ 825.1.

**Compound 5.** To a solution of compound 4 (1.24 g, 1.5 mmol) in 20 mL DCM, 3 mL TFA was added at room temperature. After 1 h, the reaction was quenched. The mixture was diluted by DCM, and washed by water, sat. NaHCO_3_. After dried by Na_2_SO_4_, the mixture was concentrated in vacuum. The residue was purified by silica column to afford compound 5 (834 mg, 83%). ^1^H-NMR (CDCl_3_ 500 MHz) 0.85 (3H, t, J=6.08 Hz), 1.06 (4H, quint, J=7.43 Hz), 1.17 (4H, quint, J=7.48 Hz), 1.28-1.37 (14H, m), 1.63 (6H, sext, J=7.00 Hz), 1.99 (4H, quint, J=8.28 Hz), 2.45 (2H, t, J=7.60 Hz), 3.26 (4H, t, J=6.83 Hz), 6.62 (1H, s), 7.27 (1H, s), 7.31 (3H, q, J=6.20 Hz), 7.60-7.63 (2H, t, J=7.35 Hz), 7.68 (2H, d, J=7.70 Hz). ^13^C-NMR (CDCl_3_ 126MHz) 14.27, 22.82, 23.62, 25.12, 27.90, 29.19, 29.43, 29.48, 29.58, 30.21, 32.04, 32.77, 34.12, 40.43, 55.12, 106.68, 117.87, 119.82, 120.08, 122.85, 127.05, 127.21, 127.89, 130.11, 138.31, 140.40, 140.95, 147.11, 150.59, 150.94, 154.46. ESI-MS: [M+H]^+^ 669.4

**Compound 6.** To a solution of compound 5 (670 mg, 1 mmol) in 20mL THF, 1.6M n-BuLi (0.75 mL, 1.2 mmol, 1.2 eq) was added at –78 ᵒC under argon. The mixture was allowed to stir for 1h at room temperature and then n-Bu_3_SnCl (488 mg, 1.5mmol, 1.5eq) was added to the mixture at 0ᵒC. After stirred at room temperature overnight, the mixture was quenched by water and was extracted by EA. The organic phase was dried by Na_2_SO_4_ and concentrated in vacuum to afford compound 6 as an oil, which was used in next step without purification.

**IR-FFC8.** Pd (PPh_3_)_2_Cl_2_ (40 mg) was added to a solution of the crude compound 7 (about 1 mmol), BBT-Br (120 mg, 0.34 mmol) and toluene (15 mL) under the inert gas atmosphere. The mixture was stirred at 110 ᵒC for 12 h. After cooling to RT, the mixture was poured into water and extracted twice with ethyl acetate. The organic phase was dried with MgSO_4_ and evaporated in vacuo. The crude material was purified by silica gel column chromatography to afford compound IR-FFC8 as dark green solid (203 mg, 39 %).

**IR-FFC8P.** IR-FFC8 (80 mg, 0.052 mmol) and sodium azide (50 mg, 0.75 mmol) were dissolved in DMF (10 mL), and the mixture was stirred for 3 h at RT. Then a large amount of water was added until all solids were dissolved. The reaction was extracted twice with ethyl acetate, the combined organic phase was dried with MgSO4 and evaporated in vacuo. The crude product was subjected to flash column chromatography on silica gel to afford the dark green solid. The dark green solid was dissolved in THF (5 mL) and copper (I) thiophene-2-carboxylate (CuTc) (5 mg), w-alkynyl-PEG-hydroxyl PEG1500 (Mn = 1500) (165 mg, about 0.11 mol) and tris[(1-benzyl-1H-1,2,3-triazol-4-yl)methyl]amine (TBTA) (3 mg) were added. The system was stirred at RT for 0.5 h, and then filtered with diatomite, and the solution was evaporated in vacuo. The crude product was purified by thin layer chromatography twice. First, ethyl acetate (EA) was used as eluant and a small amount of impurities (like the unreacted IR-BGP6) would move to the top of the TLC plate, but other parts of product 6 remained at the start point of the TLC plate. Then DCM/MeOH (10:1-5:1) was used as eluant, and the PEGylation product could be separated from alkyne-PEG. IR-BFC6P (184 mg, isolated yielding 81%) was afforded as green solid. SEC measured: Mn =5055, Mw =5426, PDI =1.07.

**Synthesis of IR-FFCHP**

**Scheme S2.** Synthetic routes of IR-FFCHP. Reagents and conditions: a) LDA, THF, -78˚C, 2h; furfural, RT, overnight; b) HCOONH_4_, 10% Pd/C, MeOH, reflux overnight; c) NBS, toluene, 0 ˚C to RT, overnight, 49%; d) n-BuLi, THF, -78˚C, argon, 1h; chloro(2,3-dimethylbutan-2-yl)dimethylsilane, argon, RT, 12h; e) n-BuLi, THF, -78˚C to RT, argon, 1h; n-Bu_3_SnCl, argon, 0 ˚C to RT, 12h; f) toluene, Pd (PPh_3_)_4_, argon, reflux, overnight, 85%; g) TFA, DCM, RT, 1h; h) n-BuLi, THF, -78˚C to RT, argon, 1h; n-Bu_3_SnCl, argon, 0 ˚C to RT, 12h; i) BBT-Br_2_, Pd (PPh_3_)_4_, toluene, reflux, overnight, 37%; j) NaN_3_,DMF, RT, 3h; k) TcCu, TBTA, w-alkynyl-PEG-hydroxyl PEG1500 (Mn = 1500), THF, RT, 30 min, 85%.

**Compound 7.** The compound 7 was prepared according to reported procedures [4].

**Compound 8.** 18.2 mL LDA (2 M, 36.4 mmol) was added into the solution of compound 7 (15 g, 36.4 mmol) in 60 mL THF at –78 ᵒC under argon. After stirred 2 h, furfural (3.5 g, 36.4 mmol) was added into above mixture. Then the mixture was stirred overnight at room temperature. After quenched by sat. NH_4_Cl, the mixture was extracted by petroleum ether. After dried by MgSO_4_, it was concentrated in vacuum to afford crude compound 8, which is used in next step without purification.

**Compound 9.** To the solution of above compound 8 in 500 mL methanol, HCOONH_4_ (7.64 g, 121.2 mmol) and Pd/C (700 mg, 10%) was added. The mixture was gently refluxed overnight, then after cooled to room temperature, the Pd/C was filtered out through a short celite column, which was washed by 200 mL petroleum ether. The mixture was washed by water three times. After dried by MgSO_4_, it was concentrated in vacuum to afford crude compound 9, which was used in next step without purification.

**Compound 10.** In a 250 mL round-bottom flask, compound 9 (3.75 g, 22.8 mmol) was dissolved in 60 mL of toluene. The flask was cooled to 0 °C using an ice-water bath. NBS (4.06 g, 22.8 mol) was added into the solution in one portion and the reaction mixture was protected from ambient light. The mixture was slowly warmed to room temperature and stirred overnight. The reaction was quenched with saturated NaHCO_3_ solution and transferred to a 500 mL of separatory funnel. The organic layer was separated and the aqueous layer was extracted two more times using diethyl ether (2 × 50 mL). The organic extracts were combined and washed with saturated NaHCO_3_ solution, dried over Na_2_SO_4_ and concentrated using rotary evaporation. The resultant crude product was dissolved in 100 mL of hexanes and filtered through a basic alumina plug to remove succinimide. The crude product was purified by distillation and the final product was obtained as a clear liquid (2.72 g, 49%). ^1^H-NMR (CDCl_3_ 400 MHz) 1.18-1.25 (4H, m), 1.43-1.50 (1H, m), 1.66-1.69 (6H, m), 2.22 (2H, d, J=7.08 Hz), 6.24 (1H, d, J=1.96 Hz), 7.35 (1H, d, J=1.96 Hz). ^13^C-NMR (CDCl_3_, 101 MHz) 26.32, 26.57, 33.01, 33.09, 38.40, 113.72, 120.63, 122.68, 143.46. ESI-MS: [M+H]^+^ 232.1

**Compound 11.** To a solution of compound 10 (3.78 g, 15.5 mmol) in 60 mL THF, 6.2 mL 2.5 M n-BuLi (18.7 mmol, 1.0 eq) was added at –78 ᵒC under argon. After stirred 1 h, chloro(2,3-dimethylbutan-2-yl)dimethylsilane (1.9 g, 16.3 mmol, 1.05 eq) was added. After stirred 12 h at room temperature, the reaction was quenched by water and extracted by DCM. The organic phase was dried by Na_2_SO_4_ and concentrated in vacuum to afford compound 11, which was used in next step without purification.

**Compound 12.** To a solution of compound 11 (4.46 g, 14.5 mmol) in 60 mL THF, 7.0 mL 2.5 M n-BuLi (17.4 mmol, 1.2 eq) was added at –78 ᵒC under argon. After stirred 1 h at room temperature, n-Bu_3_SnCl (7.08 g, 21.8 mmol, 1.5 eq) was added at 0ᵒC. After stirred 12 h at room temperature, the reaction was quenched by water and extracted by EA. The organic phase was dried by Na_2_SO_4_ and concentrated in vacuum to afford compound 12, which was used in next step without purification.

**Compound 13.** The solution of above given compound 12, 2-bromo-9,9-bis(6-bromohexyl)-9H-fluorene (7.51 g, 14.5 mmol, 1eq), Pd(PPh_3_)_4_ (660 mg, 0.73 mmol, 0.05 eq) in 50 mL toluene was refluxed overnight under argon. The solvent was removed in vacuum and the residue was purified by silica column to afford compound 13 (7.62 g, 66%). ^1^H-NMR (CDCl_3_ 500 MHz) 0.38 (6H, s), 0.65-0.66 (4H, m), 0.87-0.88 (6H, m), 0.97-0.98 (7H, m), 1.03-1.09 (4H, m), 1.17-1.26 (7H,m), 1.60-1.73 (8H, m), 1.71 (1H, t, J=10.18 Hz), 1.81 (2H, d, J=12.00 Hz), 1.97-1.99 (4H,m), 2.39-2.40 (2H, m), 3.24 (4H, t, J=6.65 Hz), 6.61 (1H, s), 7.28-7.31 (3H, m), 7.59 (1H, s), 7.67-7.68 (3H, m). ESI-MS: [M+H]^+^ 795.2

**Compound 14.** To a solution of compound 13 (2 g, 2.51 mmol) in 20 mL DCM, 3 mL TFA was added at room temperature. After 1h, the reaction was quenched. The mixture was diluted by DCM, and washed by water, sat. NaHCO_3_. After dried by Na_2_SO_4_, the mixture was concentrated in vacuum. The residue was purified by silica column to afford compound 14 (1.4 g, 85%). ^1^H-NMR (CDCl_3_ 500 MHz) 0.92-0.1.10 (4H, m), 1.04-1.10 (4H, m), 1.15-1.20 (6H, m), 1.27-1.32 (6H, m), 1.46-1.54 (1H, m), 1.64 (4H, quint, J=7.14 Hz), 1.70-1.73 (2H, m), 1.76-1.79 (2H,m), 1.96-2.00 (4H, m), 2.33 (2H, d, J=6.95 Hz), 3.26 (4H, t, J=6.83 Hz), 6.59 (1H, s), 7.25 (1H, s), 7.29-7.34 (3H, m), 7.60-7.63 (2H, m), 7.68 (2H, d, J=7.75 Hz). ESI-MS: [M+H]^+^ 653.1.

**Compound 15.** To a solution of compound 15 (772 mg, 1.2 mmol) in 20 mL THF, 1.6 M n-BuLi (0.88 mL, 1.4 mmol, 1.2 eq) was added at –78 ᵒC under argon. The mixture was allowed to stir for 1 h at room temperature and then n-Bu_3_SnCl (586 mg, 1.5 mmol, 1.5 eq) was added to the mixture at 0ᵒC. After stirred at room temperature overnight, the mixture was quenched by water and was extracted by EA. The organic phase was dried by Na_2_SO_4_ and concentrated in vacuum to afford compound 12 as an oil, which was used in next step without purification.

**IR-FFCH.** Pd(PPh_3_)_2_Cl_2_ (50 mg) was added to a solution of the crude compound 7 (about 1.2 mmol), BBT-Br (141 mg, 0.4 mmol) and toluene (15 mL) under the inert gas atmosphere. The mixture was stirred at 110 ᵒC for 12 h. After cooling to RT, the mixture was poured into water and extracted twice with ethyl acetate. The organic phase was dried with MgSO_4_ and evaporated in vacuo. The crude material was purified by silica gel column chromatography to afford compound IR-FFCH as dark green solid (221 mg, 37 %). ^1^H-NMR (CDCl_3_ 500 MHz) 1.08-1.12 (8H, m), 1.15-1.23 (8H, m), 1.33-1.42 (12H, m), 1.57-1.68 (22H, m), 1.74 (4H, d, J=12.60 Hz), 2.01-2.03 (8H, m), 2.70 (4H, d, J=6.85 Hz), 3.27 (8H, t, J=6.73 Hz), 7.02 (2H, s), 7.31-7.36 (6H, m), 7.71-7.76 (6H, m), 7.84 (2H, d, J=7.85 Hz). ^13^C-NMR (CDCl_3_ 126 MHz) 23.64, 26.41, 26.94, 27.89, 29.17, 32.75, 33.43, 34.15, 38.83, 40.41, 55.19, 110.08, 112.33, 118.41, 119.98, 120.26, 122.87, 123.57, 127.11, 127.42, 129.62, 130.79, 140.86, 141.21, 144.74, 150.73, 151.05, 152.61, 156.52. HRMS (ESI) calcd for C_78_H_90_O_2_N_4_Br_2_^81^Br_2_S_2_, ([M-H]^-^) 1498.3192, Found 1498.3217

**IR-FFCHP.** IR-FFCH (70 mg, 0.047 mmol) and sodium azide (50 mg, 0.75 mmol) were dissolved in DMF (10 mL), and the mixture was stirred for 3 h at RT. Then a large amount of water was added until all solids were dissolved. The reaction was extracted twice with ethyl acetate, the combined organic phase was dried with MgSO4 and evaporated in vacuo. The crude product was subjected to flash column chromatography on silica gel to afford the dark green solid. The dark green solid was dissolved in THF (5 mL) and copper (I) thiophene-2-carboxylate (CuTc) (5 mg), w-alkynyl-PEG-hydroxyl PEG1500 (Mn = 1500) (145 mg, about 0.94 mol) and tris[(1-benzyl-1H-1,2,3-triazol-4-yl)methyl]amine (TBTA) (3 mg) were added. The system was stirred at RT for 0.5 h, and then filtered with diatomite, and the solution was evaporated in vacuo. The crude product was purified by thin layer chromatography twice. First, ethyl acetate (EA) was used as eluant and a small amount of impurities (like the unreacted IR-BGP6) would move to the top of the TLC plate, but other parts of product 6 remained at the start point of the TLC plate. Then DCM/MeOH (10:1-5:1) was used as eluant, and the PEGylation product could be separated from alkyne-PEG. IR-BFC6P (173 mg, isolated yielding 85%) was afforded as green solid. SEC measured: Mn =4329, Mw =4558, PDI =1.05.

**3. Supplementary figures**


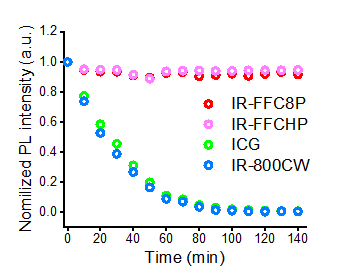


**Figure S1.** The photo-stability of fluorophores IR-FFC8P, IR-FFCHP, ICG and IR-800CW under continuous 808 nm laser irradiation for 140 min with power of 60 mW/cm^2^.


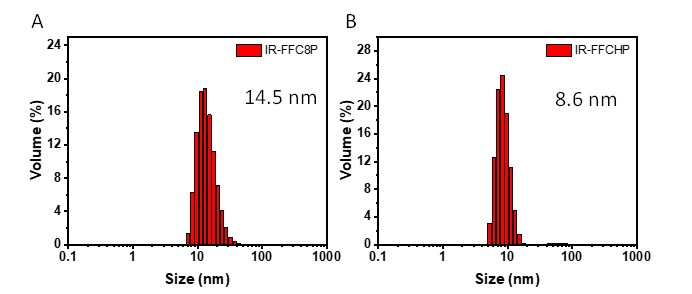


**Figure S2.** Dynamic light scattering (DLS) results of fluorophore IR-FFC8P (A) and (B) IR-FFCHP.

**Figure S3.** Calculated HOMO and LUMO of molecular fluorophores at the optimally-tuned-*ω*B97XD*/6-31G(d) level. The HOMO and LUMO energy levels are also presented in the figures. To reduce the computational requirements, long straight alkyl side chains are replaced by methyl groups. Note that the LUMO levels are obtained by subtracting the optical gap from the HOMO levels.

**Figure S4.** Optimized ground-state (S_0_) (left) and first singlet excited state (S_1_) (right) geometries of three molecular fluorophores at the *ω*B97XD*/6-31G (d) level. The dihedral angles are inserted in the figures.


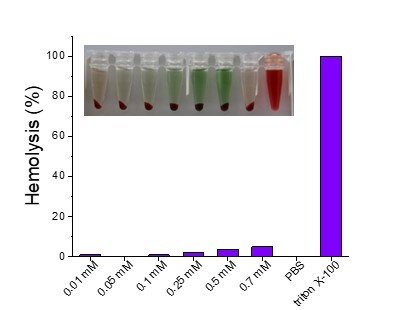


**Figure S5.** The hemolysis test result of IR-FFCHP.


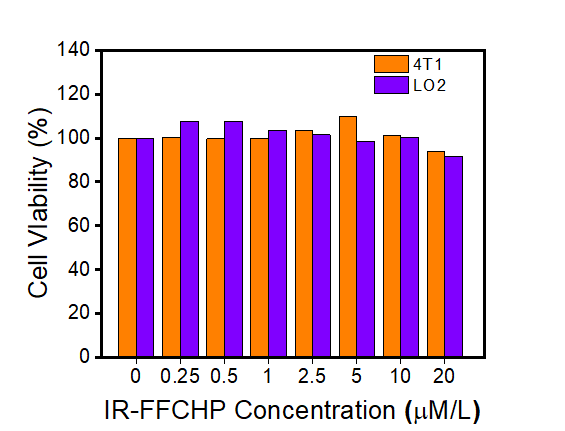


**Figure S6.** The cellular toxicity of IR-FFCHP by using 4T1 and LO2 (L-02) cell lines.

**Figure S7.** Vascular imaging of specific site in mice.

**Figure S8.** NIR-II images of hindlimb vessels in IR-FFCHP treated mice at different NIR-II long pass (LP) filters (1000-1300 nm) with different exposure times (10-700 ms).

**Figure S9.** NIR-II images of brain vessels in IR-FFCHP treated mice at different NIR-II long pass (LP) filters (1000-1300 nm) with different exposure times (10-700 ms).

**Figure S10.** The lymph nodes imaging with IR-FFCHP and PdS/CdS through different filters. Left footpad was injected with IR-FFCHP while right footpad was injected with PdS/CdS. (Left: 0.5 mM IR-FFCHP, 25 μl; right: 1.3 μM PdS/CdS-P3, 25 μL).


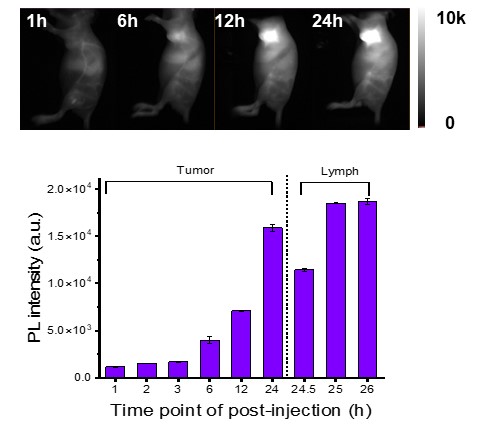


**Figure S11.** The signal accumulation in tumor after injection of IR-FFCHP in 4T1 tumor bearing mouse model, and the fluorescence signals of tumor and sentinel lymph nodes at different post-injection time points.

**Figure S12.** The hematoxylin and eosin (H&E) stain of organs in normal and 4T1 bearing mice after injection of IR-FFCHP.

**4. Supplementary tables**

**Supplementary Table 1.** Calculated first vertical *S*_0_-*S*_1_ excitation energies (*E*_01_), first vertical *S*_1_-*S*_0_ emission energies (*E*_10_), electronic configurations determined at the TD-ωB97XD*/6-31G (d) level of theory. ^a^The optimally tuned range-separated parameters included in the functionals.

| Fluorophores | ω*^a^ | *E*_01_ (*λ*_01_)  eV (nm) | f_01_ | Electronic configuration | *E*_10_ (*λ*_10_)  eV (nm) | f_10_ |
| --- | --- | --- | --- | --- | --- | --- |
| IR-FFC8 | 0.118 | 1.50 (827) | 0.5465 | HOMO → LUMO 98% | 1.20(1036) | 0.5155 |
| IR-FFCH | 0.114 | 1.43 (867) | 0.5084 | HOMO → LUMO 98% | 1.15(1074) | 0.4689 |

**Supplementary Table 2.** Retention times and molecular weights of fluorophores.

| Fluorophores | RV (mL) | M_n_  (Daltons) | M_w_  (Daltons) | M_w_/M_n_ |
| --- | --- | --- | --- | --- |
| IR-FFC8 | 16.65 | 1533 | 1575 | 1.03 |
| IR-FFC8P | 15.16 | 5055 | 5426 | 1.07 |
| IR-FFCH | 16.89 | 1241 | 1270 | 1.02 |
| IR-FFCHP | 15.48 | 4329 | 4558 | 1.05 |

**Supplementary references**

[1] M. J. Frisch, G. W. Trucks, H. B. Schlegel, G. E. Scuseria, M. A. Robb, J. R. Cheeseman, G. Scalmani, V. Barone, G. A. Petersson, H. Nakatsuji, X. Li, M. Caricato, A. V. Marenich, J. Bloino, B. G. Janesko, R. Gomperts, B. Mennucci, H. P. Hratchian, J. V. Ortiz, A. F. Izmaylov, J. L. Sonnenberg, Williams, F. Ding, F. Lipparini, F. Egidi, J. Goings, B. Peng, A. Petrone, T. Henderson, D. Ranasinghe, V. G. Zakrzewski, J. Gao, N. Rega, G. Zheng, W. Liang, M. Hada, M. Ehara, K. Toyota, R. Fukuda, J. Hasegawa, M. Ishida, T. Nakajima, Y. Honda, O. Kitao, H. Nakai, T. Vreven, K. Throssell, J. A. Montgomery Jr., J. E. Peralta, F. Ogliaro, M. J. Bearpark, J. J. Heyd, E. N. Brothers, K. N. Kudin, V. N. Staroverov, T. A. Keith, R. Kobayashi, J. Normand, K. Raghavachari, A. P. Rendell, J. C. Burant, S. S. Iyengar, J. Tomasi, M. Cossi, J. M. Millam, M. Klene, C. Adamo, R. Cammi, J. W. Ochterski, R. L. Martin, K. Morokuma, O. Farkas, J. B. Foresman, D. J. Fox, *Gaussian 16 Rev. A.03*, Wallingford, CT **2016**.

[2] a) P. J. Stephens, F. J. Devlin, C. F. Chabalowski, M. J. Frisch, *J. Phys. Chem.* **1994**, *98*, 11623; b) A. D. McLean, G. S. Chandler, *J. Chem. Phys.* **1980**, *72*, 5639; c) R. Krishnan, J. S. Binkley, R. Seeger, J. A. Pople, *J. Chem. Phys.* **1980**, *72*, 650; d) M. J. Frisch, J. A. Pople, J. S. Binkley, *J. Chem. Phys.* **1984**, *80*, 3265; e) J. Tomasi, B. Mennucci, R. Cammi, *Chem. Rev.* **2005**, *105*, 2999.

[3] C. I. Bayly, P. Cieplak, W. Cornell, P. A. Kollman, *J. Phys. Chem.* **1993**, *97*, 10269.

[4] J. Wang, R. M. Wolf, J. W. Caldwell, P. A. Kollman, D. A. Case, *J. Comput. Chem.* **2004**, *25*, 1157.

[5] W. L. Jorgensen, J. Chandrasekhar, J. D. Madura, R. W. Impey, M. L. Klein, *J. Chem. Phys.* **1983**, *79*, 926.

[6] J.-P. Ryckaert, G. Ciccotti, H. J. C. Berendsen, *J. Comput. Phys.* **1977**, *23*, 327.

[7] D. A. Case, I. Y. Ben-Shalom, S. R. Brozell, D. S. Cerutti, I. T.E. Cheatham, V. W. D. Cruzeiro, T. A. Darden, R. E. Duke, D. Ghoreishi, M. K. Gilson, H. Gohlke, A. W. Goetz, D. Greene, R. Harris, N. Homeyer, S. Izadi, A. Kovalenko, T. Kurtzman, T. S. Lee, S. LeGrand, P. Li, C. Lin, J. Liu, T. Luchko, R. Luo, D. J. Mermelstein, K. M. Merz, Y. Miao, G. Monard, C. Nguyen, H. Nguyen, I. Omelyan, A. Onufriev, F. Pan, R. Qi, D. R. Roe, A. Roitberg, C. Sagui, S. Schott-Verdugo, J. Shen, C. L. Simmerling, J. Smith, R. Salomon-Ferrer, J. Swails, R. C. Walker, J. Wang, H. Wei, R. M. Wolf, X. Wu, L. Xiao, D. M. York, P. A. Kollman, *AMBER 2018*, University of California, San Francisco **2018**.

[9] J.-D. Chai, M. Head-Gordon, *Phys. Chem. Chem. Phys.* **2008**, *10*, 6615.

[10] H. Sun, J. Autschbach, *ChemPhysChem* **2013**, *14*, 2450.

[11] H. Sun, C. Zhong, J.-L. Brédas, *J. Chem. Theory Comput.* **2015**, *11*, 3851.
